# An evidence synthesis approach to estimating the incidence of seasonal influenza in the Netherlands

**DOI:** 10.1111/irv.12201

**Published:** 2013-11-10

**Authors:** Scott A McDonald, Anne M Presanis, Daniela De Angelis, Wim van der Hoek, Mariette Hooiveld, Gé Donker, Mirjam E Kretzschmar

**Affiliations:** a Centre for Infectious Disease Control, National Institute for Public Health and the EnvironmentBilthoven, the Netherlands; b MRC Biostatistics Unit, Institute of Public HealthCambridge, UK; c Netherlands Institute for Health Services Research (NIVEL)Utrecht, the Netherlands; d Julius Centre for Health Sciences & Primary Care, University Medical Centre UtrechtUtrecht, the Netherlands

**Keywords:** Bayesian evidence synthesis, incidence, seasonal influenza, vacciation

## Abstract

**Objectives:**

To estimate, using Bayesian evidence synthesis, the age-group-specific annual incidence of symptomatic infection with seasonal influenza in the Netherlands over the period 2005–2007.

**Methods:**

The Netherlands population and age group distribution for 2006 defined the base population. The number of influenza-like illness (ILI) cases was estimated from sentinel surveillance data and adjusted for underascertainment using the estimated proportion of ILI cases that do not consult a general practitioner. The estimated number of symptomatic influenza (SI) cases was based on indirect evidence from the surveillance of ILI cases and the proportions of laboratory-confirmed influenza cases in the 2004/5, 2005/6 and 2006/7 respiratory years. In scenario analysis, the number of SI cases prevented by increasing vaccination uptake within the 65 + age group was estimated.

**Results:**

The overall symptomatic infection attack rate (SIAR) over the period 2005–2007 was estimated at 2·5% (95% credible interval [CI]: 2·1–3·2%); 410 200 SI cases (95% CI: 338 500–518 600) were estimated to occur annually. Age-group-specific SIARs were estimated for <5 years at 4·9% (2·1–13·7%), for 5–14 years at 3·0% (2·0–4·7%), for 15–44 years at 2·6% (2·1–3·2%), for 45–64 years at 1·9% (1·4–2·5%) and for 65 + years at 1·7% (1·0–3·0%). Under assumed vaccination uptake increases of 5% and 15%, 1970 and 5310 SI cases would be averted.

**Conclusions:**

By synthesising the available information on seasonal influenza and ILI from diverse sources, the annual extent of symptomatic infection can be derived. These estimates are useful for assessing the burden of seasonal influenza and for guiding vaccination policy.

## Introduction

Although infection with the influenza virus is normally self-limiting within a few weeks and most cases are uncomplicated, it is responsible for a relatively high public health and economic burden.^1^ Disease burden is primarily due to complications and excess mortality occurring in the elderly population, but also due to morbidity amongst all age groups.^2^ For the accurate estimation of the annual influenza disease burden, information on the incidence of symptomatic infection is required, which is difficult to observe and determine because only a subset of cases is normally detected using national surveillance systems.

The goal of the current study was to estimate the *symptomatic infection attack rate* (SIAR) for seasonal influenza in the Netherlands and to derive the unknown annual total number of persons with symptomatic infection (SI). The attack rate is an important epidemiological indicator of the severity of an influenza season; estimation of SIAR requires data on the number of persons with SI. Although no *direct* data exist regarding this quantity, in the Netherlands, there are a number of *indirect* sources of data – related to SI – that may be useful for estimating SI. To make best use of the available evidence, we employed Bayesian evidence synthesis, which is an established methodology for integrating all available relevant sources of evidence – both direct and indirect – to estimate a given quantity of interest.^3^,^4^

This method was applied to combine data on the annual number of persons reporting influenza-like illness (ILI), rates at which persons with ILI consult a general practitioner (GP) and the proportion of true influenza infections amongst patients with ILI. Determining the incidence of symptomatic infection, rather than of all infections, was of interest because of its relevance to the application to disease burden estimation; asymptomatic infections are not assumed to contribute to the burden of seasonal influenza.

## Methods

### Data sources

Age-specific population data for the Netherlands for 2006 were used to define the base population; data were extracted from the website of Statistics Netherlands (CBS).^5^

The annual number of cases with ILI was derived from consultations for ILI within a sentinel surveillance network of GPs. These consultation data were provided by the Continuous Morbidity Registration (CMR) Sentinel General Practice Network of NIVEL, the Netherlands Institute of Health Service Research.^6^ Annual numbers of ILI cases were stratified by age group and then averaged over the period 2005–2007. We chose a study period prior to the 2009 H1N1 pandemic because the severity and infection attack rates in 2009 differed from those for seasonal influenza.^7^ The catchment population sizes of each of the 45 sentinel GP practices were also supplied. The coverage of this nationwide sentinel network is approximately 0·8% of the population^8^ (see reference 9 for further details). The CMR data exclude those persons with ILI who do not consult a GP.

Virological testing is performed throughout the year on ILI cases, and test results were aggregated by ‘respiratory year’ (defined as week 18 of a given year through week 17 of the following year). Nose and throat swabs are sent weekly by the sentinel GP practices to the RIVM laboratory for testing by viral culture and PCR; GPs are requested to swab two ILI cases per week. The predominant influenza A strain circulating during the 2004/5, 2005/6 and 2006/7 influenza seasons was H3, although in 2005/2006 influenza B was more prevalent than A.

An Internet-based monitoring study (‘de Grote Griepmeting’, or the Great Influenza Survey, GIS) supplied data on the age-group-specific rates of GP contacts given self-reported ILI symptoms.^10^ Data from the GIS study were first averaged over the respiratory years 2004/5, 2005/6 and 2006/7 and then used to inform the adjustment for underascertainment of ILI in the Dutch sentinel surveillance system, to account for the population with ILI that do not contact a GP, and therefore were not included in the sentinel GP data.

### Evidence synthesis

Multiparameter evidence synthesis is a methodology for combining multiple sources of evidence to derive estimates for quantities that cannot easily be measured directly, typically in a Bayesian framework.^3^,^11^ By specifying a model for the relationships between the various data sources and the parameters (and related quantities) of interest, estimation can be carried out by exploiting all the available information that is directly and indirectly related to the parameters under consideration.

The Bayesian framework permits *prior* information (if available) on these parameters to be combined with observed data, to produce a *posterior* distribution, and ensures the correct propagation of uncertainty on data and parameters to the final estimates. For those parameters for which no prior information is available, vague priors are specified. Uncertainty is expressed as credible intervals, which summarise the posterior distributions of these parameters (e.g. of the size of the subpopulations and of the probability of detection (under-reporting) of cases).

**Figure 1:**
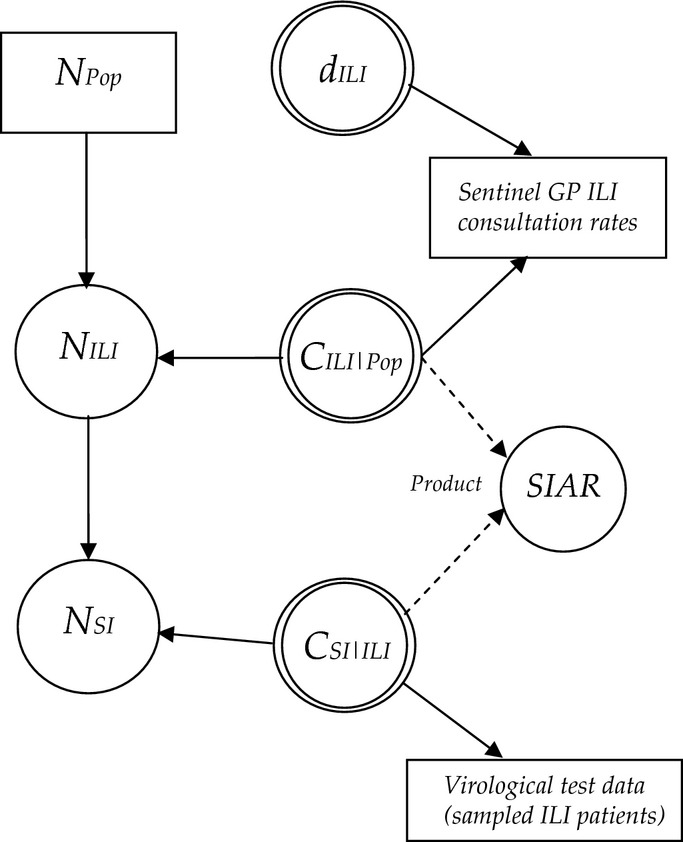
Directed acyclic graph of the relationship between model parameters and observed data; only one of the five age groups in the model is shown for clarity. Parameters include the subpopulations *N*_*ILI*_ (number with influenza-like illness) and *N*_*SI*_ (number with symptomatic infection)*,* detection probabilities (*d*), conditional probabilities (*c*) and the symptomatic infection attack rate (*SIAR*). Distributional and functional relationships are indicated by solid and dashed lines, respectively. Circles indicate model parameters. Double circles indicate parameters for which informative or vague priors are applied.

Within an evidence synthesis model, we distinguish between basic and functional parameters: basic parameters are those model parameters to which a prior distribution is assigned and functional parameters are defined as the functions of basic parameters (e.g. the conditional probability relating the number of persons with symptomatic influenza infection to the number of individuals with ILI is a basic parameter; see Fig. 1). *N* and *c*_*a|b*_ indicate the actual number of persons in a subpopulation (the number we wish to estimate) and a generic conditional probability of *a* given *b*, respectively. For example, the subpopulation of individuals with ILI, *N*_*ILI*_, is related to the total population, *N*_*Pop*_, by the conditional probability *c*_*ILI|Pop*_, and the number of individuals with SI is related to the subpopulation with ILI by the conditional probability *c*_*SI|ILI*_.

Figure 1 shows the relationship between the ‘true’ sizes of the subpopulations of interest (i.e. the actual annual number of persons with ILI (*N*_*ILI*_), and the number of symptomatically infected cases (*N*_*SI*_)), the conditional probabilities linking the true numbers corresponding to each subpopulation and the sources of direct evidence (data) informing the model parameters. Dashed arrows indicate functional relations, and solid arrows indicate distributional (i.e. stochastic) relationships. In our model (Fig. 1), data on ILI cases from sentinel surveillance inform the conditional probability *c*_*ILI|Pop*_, and data from virological testing inform the conditional probability *c*_*SI|ILI*_.

### Model specification

Parameters for five separate age groups (<5 years, 5–14 years, 15–44 years, 45–64 years and 65+ years) were estimated whenever age-group-specific data or age-group-specific prior information was available. The task is to compute the posterior distribution of all parameters, including the subpopulations (*N*_*ILI*_*, N*_*SI*_) (Fig. 1).

#### Basic parameters

Basic parameters (see Table 1, in the format of reference^4^) include parameters relating the numbers in each subpopulation, and conditional and detection probabilities. We assume that the actual numbers in the subpopulations *N*_*ILI*_ and *N*_*SI*_ are binomially distributed.

**Table 1:** Model parameters, the specified prior distribution or functional form, and evidence (direct or indirect, or both) informing the parameter

Parameter	Distribution/functional form	Rationale	Evidence
*c*_*a,ILI|Pop*_	Beta(1,1)	Vague prior, assuming nothing is known about this parameter	Indirect evidence from observed ILI cases (GP consultations) from sentinel surveillance
*c*_*a,SI|ILI*_	Beta(1,1)	Vague prior, assuming nothing is known about this parameter	Direct evidence from virological testing performed on random samples of ILI cases, age group specific
*d*_*a,ILI*_	Beta(1,1)	Vague prior, assuming nothing is known about this parameter	Data from the GIS study (Friesma *et al*., 2009)^10^: age-group-specific numbers of self-reported ILI cases and ILI cases who visited a GP.
*N*_*a,Pop*_	N/A	N/A	CBS population estimates for 2006
*N*_*a,ILI*_	*Binomial(N_a,Pop,ca,_ILI_Pop_)*	Binomial likelihood of the ‘true’ number of ILI cases, with parameters population size and the proportion with ILI	Observed sentinel data on ILI cases; binomial likelihood for observed data with detection probability *d*_*a,ILI*._
*N*_*a,SI*_	*Binomial(N_a,ILI_,c_a,SIILI_)*	Binomial likelihood of the ‘true’ number of SI cases, with parameters the number of ILI cases and the proportion with SI	All model assumptions and data
*SIAR*_*a*_	*c*_*a,SI|ILI*_ × *c*_*a,ILI|Pop*_	Symptomatic infection attack rate is the product of component conditional probabilities	All model assumptions and data

*N*_*a,ILI*_ ˜ *Binomial*(*N*_*a,Pop*_, *c*_*a,ILI|Pop*_)
*N*_*a,SI*_ ˜ *Binomial*(*N*_*a,ILI*_, *c*_*a,SI|ILI*_)
*c*_*a,ILI|Pop*_ Probability of ILI in the population
*c*_*a,SI|ILI*_ Probability of symptomatic influenza infection given ILI
*d*_*a,ILI*_ Proportion of true ILI cases that are reported

The prior distributions for these parameters are specified below.

#### Functional parameters

The SIAR is the only functional parameter to be estimated and is expressed as the product of age-group-specific conditional probabilities.


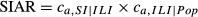


#### Size of relevant subpopulations: priors and distributional assumptions

##### Influenza-like illness

The probability of ILI occurring in the population, *c*_*a,ILI|Pop*_ (the ILI attack rate), is informed by data on the annual number of patients with ILI provided by the sentinel GP surveillance data (a sample of all ILI cases) and the estimated underascertainment of actual ILI cases. The number of sampled ILI cases, *y*_a,GP_, was assumed to be binomially distributed, with priors on the detection probability *d*_*a,ILI*_ constructed from age-group-specific ascertainment estimated from the Internet-based monitoring (GIS) study. Ascertainment was defined according to the age-group-specific number of (self-reported) ILI cases in the GIS study, *n*_*a,GIS*_, and the number of ILI cases from the same study who reported visiting a GP, *y*_*a,GIS*_. Point estimates of the proportions of ILI cases reporting having visited a GP were 0·240, 0·243, 0·196, 0·220 and 0·309 for the age groups <5 years, 5–14 years, 15–44 years, 45–64 years and 65+ years, respectively. A vague prior, *Beta*(1,1), was specified for the ascertainment parameter *d*_*a,ILI*_. Data on the number of patients with ILI captured by the sentinel GP network, *y*_*a,GP*_, and the size of the sentinel population, *n*_*a,GP*_, were used directly, together with estimated ascertainment to account for those ILI cases not consulting a GP, to inform the conditional probability *c*_*a,ILI|Pop*_.

*y*_*a,GIS*_ ˜ *Binomial(n*_*a,GIS*_*, d*_*a,ILI*_)
*y*_*a,GP*_ ˜ *Binomial(n*_*a,GP*_*, d*_*a,ILI*_ *× c*_*a,ILI|Pop*_)

#### Symptomatic infection

The conditional probability of being infected with influenza given ILI symptoms is directly informed by data from annual virological testing of samples provided by the sentinel GPs, for which the number of positive cases by age group *a* and per respiratory year *t*, *y*_*a,t,VIR*_, and the denominator (ILI samples tested), *n*_*a,t,VIR*_, were available. This probability also depends on the sensitivity of the test, *π,* which was assigned a uniform prior ranging from 95% to 100%. The test sensitivity was incorporated into the binomial likelihood for the observed number of positive samples *y*_*a,t,VIR*_. Positivity rates over the respiratory years 2004/5, 2005/6 and 2006/7 were on average 25·9%, 41·5%, 30·7%, 26·3% and 25·0% for the age groups <5 years, 5–14 years, 15–44 years, 45–64 years and 65+ years, respectively. A vague prior, *Beta*(1,1), was specified for the conditional probability *c*_*a,SI|ILI*_.

*π ˜ Uniform*(0·95,1)
*y*_*a,t,VIR*_ ˜ *Binomial(n*_*a,t,VIR*_*, π × c*_*a,SI|ILI*_)

## Scenario analysis

A scenario analysis was conducted to examine the effect of increasing vaccination uptake amongst the 65 + age group (estimated at >70% within this age group in the period 1998–2005 and at >75% in 2008–2010^12^,^13^) to 80%, 85% and 90% (assuming uptake was on average 75% in the period 2005–2007), by quantifying the expected number of SI cases prevented. The effect of increasing vaccination uptake was crudely simulated by adjusting the data – specifically, the number of sentinel-reported ILI cases in the over-65-year-olds – so that the expected ILI incidence rate (not taking into account underascertainment) decreased by 1·7/10 000 per percentage increase in simulated vaccination uptake. This parameter estimate (1·7/10 000) was based on the results of a linear regression of sentinel-based ILI incidence rates on vaccine uptake conducted by RIVM.^9^

## Model inference

**Figure B1:**
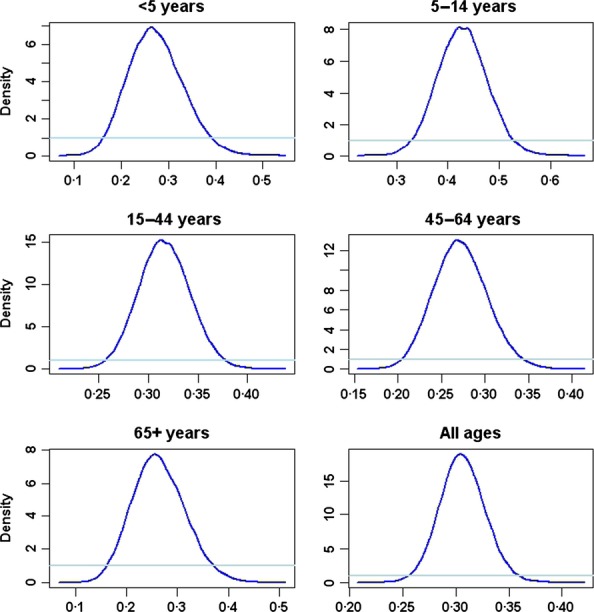
Posterior and prior distributions for the parameter *c*_*SI*__*|ILI*_ for the five age groups and the all-ages category. The posterior and prior are, respectively, indicated with dark blue and light blue lines.

Sampling from the posterior distributions for each parameter was carried out via MCMC simulation using OpenBUGS, version 3·2·1^14^ and the BRugs package for R.^15^ Two independent chains were run for 80 000 iterations, with the first 30 000 iterations ignored (treated as burn-in). Brooks–Gelman–Rubin diagnostic plots were examined to check that convergence of the chains was satisfactorily achieved. Posterior estimates were produced for 26 stochastic nodes (5 parameters x 5 age groups, plus test sensitivity *π*) and for 5 functional nodes (1 parameter x 5 age categories). Figure B1 (Appendix B) shows the overlaid prior and posterior distributions for an example parameter.

## Results

**Figure 2:**
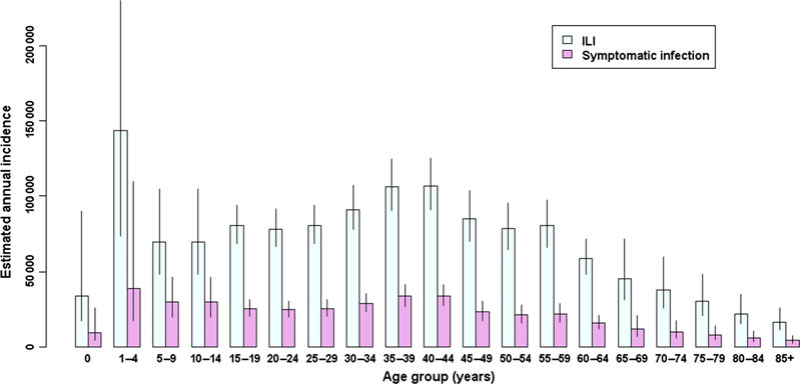
Posterior median (with 95% credible interval) estimates of the annual number of incident cases of influenza-like illness (*N*_*ILI*_) and of symptomatic infection (*N*_*SI*_), stratified by age group.

Tables2 and 3 show the posterior summaries for the various basic and functional parameters by age group and the estimated numbers in each level of the population. The estimated SIAR was highest for the <5 years group (4·9%, 95% credible interval (CI): 2·1–13·7%) and lowest for the 65+ years group (1·7%, 95% CI: 1·0–3·0%). An annual total number of 410 200 SI cases (95% CI: 338 500–518 600) was estimated for the Netherlands. Figure 2 shows the estimated annual number of ILI and SI cases, stratified by the 5-year age group.

**Table 2:** Posterior median (95% credible interval) estimates of SIAR, *c*_*ILI|Pop*_ and *c*_*SI|ILI*_, stratified by age group

Age group	SIAR (95% CI)	*c*_*ILI|Pop*_ (95% CI)	*c*_*SI|ILI*_ (95% CI)
<5 years	4·9% (2·2–13·7%)	0·180 (0·093–0·482)	0·271 (0·168–0·395)
5–14	3·0% (2·0–4·7%)	0·070 (0·050–0·103)	0·428 (0·334–0·526)
15–44	2·6% (2·1–3·2%)	0·081 (0·071–0·094)	0·316 (0·267–0·368)
45–64	1·9% (1·4–2·5%)	0·070 (0·059–0·083)	0·271 (0·215–0·335)
65+	1·7% (1·0–3·0%)	0·065 (0·046–0·102)	0·261 (0·169–0·372)
Total	2·5% (2·1–3·2%)	0·082 (0·072–0·101)	0·306 (0·265–0·350)

SIAR, symptomatic infection attack rate; ILI, influenza-like illness; Pop, population; SI, symptomatic infection; CI, credible interval.

**Table 3:** Posterior median estimates of the annual numbers of incident cases of ILI and SI (*N*_*ILI*_ and *N*_*SI*_, respectively), stratified by age group

Age group	*N*_*ILI*_ (95% CI)	*N*_*SI*_ (95% CI)
<5 years	177 400 (90 890–477 500)	48 200 (20 240–137 600)
5–14	139 600 (96 980–209 800)	59 560 (36 230–96 900)
15–44	543 900 (464 400–637 800)	171 800 (132 200–219 300)
45–64	303 300 (249 600–368 900)	82 340 (58 250–112 900)
65+	151 900 (104 600–239 700)	39 710 (21 350–71 930)
Total	1 336 000 (1 169 000–1 654 000)	410 200 (338 500–518 600)

ILI, influenza-like illness; SI, symptomatic infection; CI, credible interval.

The effects of the three increased vaccination uptake scenarios are shown in Table 4. With an increase of 5% in persons aged 65+ years, a projected 1970 annual cases of SI would be prevented (5·0% of the SI cases in the baseline scenario). With an increase in vaccine uptake of 15%, an estimated 5310 SI cases would be prevented in this age group (13·4% of the baseline SI cases).

**Table 4:** Results of the vaccination uptake scenario analysis: posterior median (with 95% credible interval) estimates of the annual number of incident SI cases *(N*_*SI*_) and point estimates of the number of SI cases averted in persons aged 65 + years (compared with baseline of 0% increase in uptake)

Simulated increase in vaccine uptake	*N*_*SI*_ (95% CI)	Averted cases
0% (baseline)	39 710 (21 350–71 930)	–
5%	37 740 (20 230–68 690)	1970
10%	36 180 (19 160–65 100)	3530
15%	34 400 (18 210–62 390)	5310

SI, symptomatic infection; CI, credible interval.

## Discussion

By synthesising a number of national data sources on ILI and influenza, we estimated an overall SIAR of 2·5% for symptomatic infection with influenza in the Netherlands in the period 2005–2007. The estimated SIAR varied by age group and was largest for children under 5 years of age (4·9%; *N*_SI_ of 48 200) and smallest for adults 65 years and older (1·7%; *N*_SI_ of 39 710).

We suggest that the current evidence synthesis model is of value for projecting the consequences of improved public health interventions. Influenza vaccination coverage of the elderly in the Netherlands is amongst the highest in Europe;^16^ the results of the vaccination uptake scenario analysis suggested that even a small increase in vaccination uptake (of 5%) within the 65 + age group would have a visible impact on the expected disease burden for influenza, with a predicted 1970 SI cases potentially averted. Projections are conservative, however, because the potential for reduced transmission rates expected with increasing vaccination coverage was not considered. Even without improvements in vaccination uptake, the burden of influenza is expected to increase due to demographic change.^17^

There are few previous studies that have derived the incidence of influenza in the Netherlands. The Dutch Public Health Status and Forecast Studies (DPHSF) used an estimated influenza incidence of 336 per 10 000 people in their calculation of disease burden for 2007.^18^ This corresponds to an attack rate of 4·8%, higher than our estimated overall attack rate. Although the DPHSF estimate adjusted for the proportion of influenza/ILI cases visiting a GP (30%), it did not adjust for the proportion of ILI that were true influenza cases. Another study positions the attack rate lower, at 1–2% over the period (1997/8 to 2006/7), but this is based on ILI consultation rates only,^19^ which is only one of the data sources synthesised in the current model.

Bayesian evidence synthesis methods have a number of advantages, in that diverse sources of information – both in the form of data and as prior distributions – can be combined in a coherent framework, and uncertainty around model parameters is correctly propagated through the model to the final estimates. In addition, incomplete or missing information regarding certain parameters, such as for a subset of age groups/strata considered, can be accommodated by specifying vague prior distributions.

We also explored a version of the model including an additional subpopulation, those patients hospitalised due to influenza; results are described in Appendix A. This model variant was not retained because a lack of age-group-specific evidence to inform the conditional probability of hospitalisation given SI entailed an assumption that this probability was equal across age groups, which conflicted with the age-specific evidence on *N*_SI_, particularly for the youngest age group. This illustrates that biased estimates of the parameters of interest can be obtained when the available evidence is insufficient or in conflict.^20^

The validity of the estimates derived here, as for any estimation method, is dependent on the assumptions made when constructing the model and the quality (representativeness and bias) of the available data. Children and elderly persons are under-represented in the Internet-based monitoring study, but are the age groups most likely to consult a GP with ILI; the degree of ascertainment may be underestimated for these age groups, leading to overestimation of ILI and SI incidence. In addition, the number of true ILI cases may be larger than the number computed from the proportion of GIS survey participants who reported visiting their GP (because the likelihood of consulting a GP is associated with symptom severity, the CMR sentinel surveillance system is less likely to capture the less-severe ILI cases), and consequently, the incidence of both ILI and SI would be underestimated. In addition, some self-reported ILI cases may not be considered ILI by a GP, because the GIS' defining symptom set for ILI (sudden fever onset and muscle pain, and cough and/or sore throat and/or chest pain) is consistent with other infectious agents.

Other limitations concern representativeness over age groups in the sentinel surveillance data due to differential healthcare-seeking behaviour. Working age adults may be less likely than other age groups to have a GP consultation (as suggested by the self-reported proportions from the GIS study) despite having relatively severe ILI, due to time constraints and/or the knowledge that influenza is self-limiting. In this instance, the sentinel data may under-represent working age adults, and thus, SIAR and SI incidence would be correspondingly underestimated. Such health-seeking behaviour is country specific as well as age dependent; factors associated with the health care and social security systems can influence the likelihood of GP consultation. In some countries, GP consultation is required to legitimate sick leave, but this is not so in the Netherlands. Such a social security condition may highly influence the number of GP consultations amongst working persons.

We applied evidence synthesis methods to the period 2005–2007 and averaged ILI data over these three years, as our goal was to estimate symptomatic incidence during a typical influenza season. However, this removes real between-season variability in incidence; influenza seasons differ with respect to timing and the number of other respiratory viruses circulating. We estimated incidence prior to the 2009 H1N1 pandemic because the severity and infection attack rates, and the age-dependent pattern of attack rates differed substantially from seasonal influenza attack rates in 2009.^7^

In conclusion, using Bayesian evidence synthesis techniques, we have combined a variety of national data sources to derive robust estimates for the critical epidemiological parameters – SIAR and SI incidence – required for the estimation of the health burden of symptomatic influenza in the Netherlands. Uncertainty in these estimates, given as 95% credible intervals, reflects the quantity and quality of the available data sources.

## Figures and Tables

**Figure A1:**
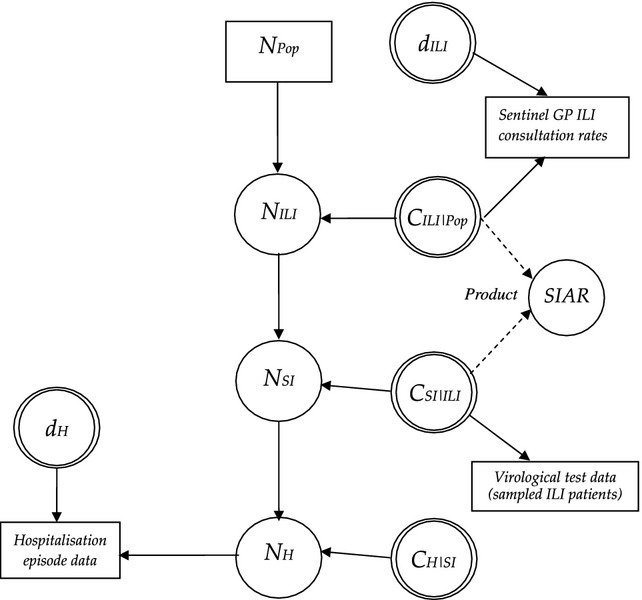
Directed acyclic graph of the relationship between model parameters and observed data, additionally including nodes for influenza-related hospital episode data, the size of the actual hospitalised subpopulation (*N*_*H*_) and the detection probability (*d*_*H*_).

## Appendix A

In this appendix, we describe the results of additionally incorporating data on patients hospitalised for influenza and associated parameters in the evidence synthesis model.

Hospitalised cases within the period 2005–2007 were extracted from the National Medical Registration (NMR), an inpatient hospitalisation database maintained by PRISMANT, where an influenza-related hospital episode was defined as a hospital admission with mention of one or more of the ICD-9 diagnosis codes 487, 487·0, 487·1, 487·8; counts were divided into five age groups and averaged over the period 2005–2007.

In this model variant, the observed and estimated numbers of influenza-associated hospitalisations (*y*_*a,NMR*_ and *N*_*H*_, respectively) are incorporated. We added the following basic parameters and relationships (see Fig. 1A):

*c*_*a,H|SI*:_ Probability of hospitalisation given symptomatic infection
*d*_*H*_: Proportion of all influenza-related hospital admissions that are registered
*N*_*a,H*_ ˜ *Binomial(N*_*a,SI*_*, c*_*a,H|SI*_): Relationship between influenza-related hospitalisations and symptomatic infections
*y*_*a,NMR*_ ˜ *Binomial(N*_*a,H*_, *d*_*H*_): Relationship between registered influenza-related hospital episodes and detection probability

The number of influenza-related cases in hospital is assumed to be under-reported, with detection probability *d*_*H*_ constrained to be equal for all age groups. The vague prior *Beta*(1,1) was specified for *d*_*H*_, as no information was available on this parameter. The conditional probability of being hospitalised given symptomatic infection (*c*_*a,H|SI*_) for all age groups was assigned the prior *Beta*(10 7004), which corresponds to the proportion (0·14%, 95% CI: 0·095–0·27) of influenza cases during the 2009 H1N1 pandemic that were hospitalised in the Netherlands.^7^

Posterior median estimates of the *N*_*ILI*_, *N*_*SI*_ and *N*_*H*_ subpopulations are shown in Table 1A. The estimated median number of incident SI cases within the youngest age group (<5 years), 107 300, is 2·2-fold larger than the estimated SI incidence derived from the evidence synthesis excluding hospitalisation (48 200). Consequently, both the overall SIAR (2·7%) and the SIAR estimated for the youngest age group (10·9%) were higher than those estimated from the model without an H layer.

**Table A1:** Posterior median (95% credible interval) estimates of the annual numbers of incident cases of ILI, SI and hospitalisations, stratified by age group

Age group	*N*_*ILI*_ (95% CI)	*N*_*SI*_ (95% CI)	*N*_*H*_ (95% CI)
<5 years	269 800 (175 300–323 600)	107 300 (62 340–149 000)	171 (115–251)
5–14	115 200 (83 730–144 500)	42 250 (32 550–570 30)	43 (27–67)
15–44	525 900 (478 200–589 000)	143 700 (125 200–181 200)	112 (73–167)
45–64	283 800 (246 200–320 800)	74 600 (61 310–94 940)	80 (52–119)
65+	190 700 (131 500–262 300)	68 460 (43 530–84 950)	137 (93–199)
Total	1 357 000 (1 258 000–1 522 000)	433 500 (397 700–474 000)	547 (369–774)

The reason for the discrepancy in estimated *N*_*SI*_ and SIAR is due to the lack of evidence informing the prior parameters *d*_*a,H*_ and the lack of age-group-specific evidence informing the conditional probability of being hospitalised given symptomatic infection, *c*_*a,H|SI*_. The latter prior distribution was specified based on a study of the H1N1 pandemic, the severity of which may not be comparable with the influenza strains circulating during the study period. Because the detection probability *d*_*H*_ was not identified by any evidence sources and the conditional probability *c*_*H|SI*_ was a priori specified as equal across all age groups (although intuitively *c*_*H|SI*_ should be age-dependent; i.e. much lower for children than for the elderly), many more hospitalised cases for the <5 years age group were postulated (*n* = 171) than were observed (*n* = 88). This top-down information unduly influenced the posterior distribution for SI for this age group, and consequently the SIAR.

## Appendix B

In this appendix, we present a supplementary plot to graphically compare prior and posterior distributions for an example model parameter (Fig. 1B).

## References

[1] Molinari NA, Ortega-Sanchez IR, Messonnier ML (2007). The annual impact of seasonal influenza in the US: measuring disease burden and costs. Vaccine.

[2] Meier CR, Napalkov PN, Wegmuller Y, Jefferson T, Jick H (2000). Population-based study on incidence, risk factors, clinical complications and drug utilisation associated with influenza in the United Kingdom. Eur J Clin Microbiol Infect Dis.

[3] Presanis AM, De Angelis D, Hagy A (2009). The severity of pandemic H1N1 influenza in the United States, from April to July 2009: a Bayesian analysis. PLoS Med.

[4] Presanis AM, Pebody RG, Paterson BJ (2009). Changes in severity of 2009 pandemic A/H1N1 influenza in England: a Bayesian evidence synthesis. BMJ.

[5] Statistics Netherlands (CBS) (2012). The Population of the Netherlands by Age and Sex.

[6] Donker GA (2012). Continuous Morbidity Registration at Dutch Sentinel General Practice Network 2011.

[7] Steens A, Waaijenborg S, Teunis PF (2009). Age-dependent patterns of infection and severity explaining the low impact of 2009 influenza A (H1N1): evidence from serial serologic surveys in the Netherlands. Am J Epidemiol.

[8] Donker GA (2011). Continuous Morbidity Registration Sentinel Stations the Netherlands 2010.

[9] Dijkstra F, Donker GA, Wilbrink B, Van Gageldonk-Lafeber AB, Van Der Sande MA (2009). Long time trends in influenza-like illness and associated determinants in The Netherlands. Epidemiol Infect.

[10] Friesema IH, Koppeschaar CE, Donker GA (2009). Internet-based monitoring of influenza-like illness in the general population: experience of five influenza seasons in The Netherlands. Vaccine.

[11] Presanis AM, De Angelis D, Goubar A, Gill ON, Ades AE (2011). Bayesian evidence synthesis for a transmission dynamic model for HIV among men who have sex with men. Biostatistics.

[12] Statistics Netherlands (CBS) (2011). Influenza Vaccination by Age, Sex And Risk Group.

[13] Jansen B, Tacken M, Mulder J, Visscher S, Tiersma W, Braspenning J (2011). Monitoring Vaccination Rates: Dutch National Influenza Prevention Programme 2010.

[14] Lunn D, Spiegelhalter D, Thomas A, Best N (2009). The BUGS project: Evolution, critique and future directions. Stat Med.

[15] Thomas A, O'Hara B, Ligges U, Sturtz S (2006). Making BUGS Open. R News.

[16] Mereckiene J, Cotter S, D'Ancona F (2010). Differences in national influenza vaccination policies across the European Union, Norway and Iceland 2008-2009. Euro Surveill.

[17] McDonald SA, van Lier A, Plass D, Kretzschmar ME (2012). The impact of demographic change on the estimated future burden of infectious diseases: examples from hepatitis B and seasonal influenza in the Netherlands. BMC Public Health.

[18] Hoeymans N, Gommer AM, Poos MJJC (2007). Mortality, Morbidity and Disease Burden for 56 Selected Disorders.

[19] Dijkstra F, van Gageldonk-Lafeber AB, Brandsema P (2007). Annual Respiratory Diseases Report 2006/2007.

[20] Presanis A, Spiegelhalter DJ, Seaman S, Goubar A, Ades AE (2008). Conflicting evidence in a Bayesian synthesis of surveillance data to estimate human immunodeficiency virus prevalence. J R Stat Soc Ser A Stat Soc.

